# IL-10, IL-15, IL-17, and GMCSF levels in cervical cancer tissue of Tanzanian women infected with HPV16/18 vs. non-HPV16/18 genotypes

**DOI:** 10.1186/s13027-015-0005-1

**Published:** 2015-03-20

**Authors:** Adriana C Vidal, David Skaar, Rachel Maguire, Seyram Dodor, Laura W Musselwhite, John A Bartlett, Olola Oneko, Joseph Obure, Pendo Mlay, Susan K Murphy, Cathrine Hoyo

**Affiliations:** Department of Surgery, Division of Urology, Cedars-Sinai Medical Center, Los Angeles, CA USA; Department of Biological Sciences, Center for Human Health and the Environment, North Carolina State University, Raleigh, NC USA; School of Science and Mathematics, North Carolina State University, Raleigh, NC USA; Global Health Program, Duke University Medical Center, Durham, NC USA; Department of Medicine, Division of Infectious Diseases, Duke University School of Medicine, Durham, NC USA; Kilimanjaro Christian Medical Center, Moshi, Tanzania; Department of Obstetrics and Gynecology, Division of Gynecologic Oncology, Duke University School of Medicine, Durham, NC USA

**Keywords:** Invasive cervical cancer, Case-only, HPV infection, Cytokines, Race, Disparities

## Abstract

**Background:**

Despite comparable screening rates for precancerous lesions, higher incidence and mortality related to cervical cancer in minority women persists. Recent evidence suggests that minority women with precancerous cervical lesions harbor a wider range of human papillomavirus (HPV) genotypes, many of these distinct from HPV16/18, those most commonly found in Caucasian women. The goal of the analysis was to determine if inflammatory cytokines and chemokines varied by HPV 16/18 versus other genotypes in cervical cancer tissues from Tanzanian women.

**Methods:**

HPV genotypes and concentrations of chemokines and cytokines were measured from homogenized fresh tumor tissue of thirty-one women with invasive cervical cancer (ICC). Risk factors for cervical cancer including age, parity, hormonal contraceptive use and cigarette smoking were obtained by questionnaire. Generalized linear models were used to evaluate differences between chemokines/cytokine levels in women infected with HPV16/18 and those infected with other HPV genotypes.

**Results:**

After adjusting for age, parity and hormonal contraceptives, IL-17 was found significantly more frequently in invasive cervical cancer samples of women infected with HPV16/18 compared to women infected with other HPV genotypes (p = 0.033). In contrast, higher levels for granular macrophage colony-stimulating factor (p = 0.004), IL-10 (p = 0.037), and IL-15 (p = 0.041) were found in ICC tissues of women infected with genotypes other than HPV16/18 when compared to those of women infected with HPV16/18.

**Conclusions:**

While the small sample size limits inference, our data suggest that infection with different HPV genotypes is associated with distinct pro-inflammatory cytokine expression profiles; whether this explains some of the racial differences observed in cervical cancer is still unclear. Future studies are needed to confirm these findings.

**Electronic supplementary material:**

The online version of this article (doi:10.1186/s13027-015-0005-1) contains supplementary material, which is available to authorized users.

## Background

Despite major reductions in incidence and mortality over the last decade, an estimated 12,340 cases of invasive cervical cancer (ICC) continue to be diagnosed in the United States annually, with the highest incidences in Hispanic and African American women when compared to European American women [1]. Liquid-based cytology screening rates, whether self-reported or estimated from insurance claims data, accounted for most of the overall decline in incidence and mortality, but are comparable among African Americans, Hispanics and European Americans, and thus fail to explain the racial/ethnic discrepancies in ICC incidence and mortality [2-4].

Reasons for continued racial/ethnic disparities in ICC incidence and mortality are still unclear, although published data in the last few years suggest that among women with precancerous lesions, African Americans may harbor human papillomavirus (HPV) genotypes distinct from those found in European American women [5-7]. However, these studies are based on high grade lesions and do not include invasive cancer cases, and in general, analyses are conducted in cells obtained from cervical scrapes. We previously found among African women that HPV 31, 35, and 45 in addition to HPV 16 and 18, were the genotypes most frequently found in ICC cases [8]. While HPV 16 was found in 73% of the tissue samples in those ICC cases with multiple infections, and HPV 35 and 45 were found in 33%, and HPV 18 in 21% of ICC tissue samples, it is possible that co-infection with high-risk (HR)-HPV 35 and 45 may have also influenced progression to ICC in these women. Nonetheless, these findings raise the possibility that different immune responses to persistent infection with distinct HR-HPV genotypes may explain some of the ICC racial/ethnic disparities observed worldwide [9].

It is known that HR-HPV initiates local Th2 inflammation at an early stage, involving antibody forming cells, which fosters an immunosuppressive microenvironment that aids tumor progression [10]. Indeed, Paradkar et al. [11], have proposed using cytokine level measurements as biomarkers for risk-stratification of patients with precancerous lesions. Increased IL-17 expression is associated with cervical cancer cell growth along with IL-6 levels in tumor tissues; IL-17 may act through IL-6 [11]. In addition, increased levels of IL-10, one of the best-studied Th2 type cytokines which has a general immunosuppressive function [12], has been associated with risk of high-grade cervical lesions [13-15]. Furthermore, Scott et. al. [16] reported that significantly increased levels of MIP-1a, TNF, IL-12 and IL-10 cytokines were associated with a reduced likelihood of any HPV clearance (including low and high-risk types) among women with incident HPV infections, but without cervical intraepithelial neoplasia (CIN).

In this analysis we examined a wide spectrum of cytokines and chemokines present in ICC tissues from Tanzanian patients to determine if cytokine levels differed by HPV genotype (HPV 16 or 18 vs. other HPV genotypes).

## Methods

### Study participants

Study procedures were approved by Research Ethics Boards at KCMC and Duke University School of Medicine Institutional Review Board, and all study participants signed an informed consent explaining the nature of the study. The patients included in the present analyses were part of a case–control study aimed at identifying epigenetic marks (DNA methylation and gene expression profiles) associated with progression from CIN-1 to invasive cancer. Detailed methods of patient identification and enrollment have been described elsewhere [8,17]. Briefly, between November 2008 and March 2009, eligible study participants were identified from the appointment books of the Reproductive Health Clinic (RHC) at Kilimanjaro Christian Medical Centre (KCMC), a Cervical Cancer prevention clinic funded by the World Health Organization (http://www.afro.who.int/en/tanzania/who-country-office-tanzania.html). KCMC is a tertiary care facility that serves a catchment area of ~10 million individuals. Eligible participants were 18 years or older and had no history of an abnormal Pap test. ICC patients comprised new cases with no history of cervical abnormalities who were also 18 years or older and were referred for colposcopic directed evaluations. A trained nurse interviewer enrolled a total of 249 women; all but 2 approached agreed to participate (99% response rate). Of these, 12 patients were excluded due to missing or inadequate Pap smear, refusal of an HIV-1 antibody test, and diagnosis of an unrelated co-morbid condition. The final number of participants, n = 215 (86%), included participants who completed questionnaire, and the availability of CIN status, and HPV genotype data. Among the n = 215 participants, n = 40 were histologically confirmed ICC cases and n = 37 were further genotyped for any HPV infection. Of these n = 37, we excluded 4 cases that did not harbor HPV infection and 2 cases with insufficient tissue available to homogenize. Thus, thirty-one women had adequate tissue to successfully measure cytokine/chemokine profiles from homogenized tissue.

### Data collection

A trained nurse-interviewer obtained informed consent from all participants, and administered a standardized 40-minute questionnaire in person. The questionnaire collected information on socio-demographic characteristics (e.g., age, marital status), type of marriage (polygamy vs. monogamy), tribe, educational attainment, cigarette smoking, alcohol intake, reproductive history (e.g., menarche, parity and gravidity), sexual history (e.g., lifetime number of sexual partners, age at first intercourse), and medication and supplement use.

### Specimen collection and handling

Biopsies of lesions were obtained during colposcopy for all cancers for histological evaluation as part of routine patient care, and ICC was diagnosed by Hematoxilin and Eosin staining. A portion of tumor samples was aliquoted and immediately placed on dry ice, for research purposes. Following specimen collection, routine cervical visual inspection was also performed and treatment conducted according to standard clinical protocol.

#### Pathologic diagnosis of CIN and Carcinoma

Papanicolaou smears and biopsy specimens were processed and read by the staff pathologist at KCMC using standard conventions according to ASCCP guidelines as appropriate (http://www.asccp.org/). Once a month, medical charts were reviewed by KCMC staff pathologist for HIV-1 test and cyto-pathological results, to classify cases using the Bethesda classification system [18]. Based on pathology and medical records findings, results were then coded as “no evidence of cytological abnormality”, “mild dysplasia” including LSIL and CIN1, “moderate dysplasia” including HSIL and CIN2-3, or “cancer” which included squamous cell carcinoma and two adeno-squamous carcinomas of the uterine cervix. None of the specimens were read as “atypical cells of uncertain significance (ASCUS)”. These results were available as part of clinic records, and pathologists entered them into the database. These clinical results were then compiled and transferred according to protocol.

#### HPV genotyping

ThinPrep® specimens and homogenized aliquoted biopsies collected during the same visit were shipped to the University of Hawaii Cancer Center. Following DNA extraction, PGMY09/PGMY11 primers [19,20] were used in PCR to target a 450-bp region of the HPV L1 genome. Amplification of the human β-globin gene was included as an internal control for sample sufficiency. All specimens were suitable for viral DNA analysis. HPV-positive specimens were subsequently genotyped by using the HPV Linear Array® (Roche Molecular Systems Inc., Branchburg, NJ, USA).

#### Cytokine measurements

Thirty-one tumor tissues were homogenized in Tissue Extraction Reagent I (Life Technologies/Invitrogen, Grand Island, NY) using a Qiagen TissueRuptor (Qiagen, Valencia, CA), centrifuged to pellet debris, and the supernatant removed and frozen at −80°C. Frozen samples were shipped to the ProteoGenomics Facility at the Medical University of South Carolina on dry ice, for analysis using the Human Cytokine Magnetic 30-Plex panel (IL-15, IL-4, IL-2, IL-1RA, IL-1B, IL-5, IL-6, IL-7, IL-8, EGF, IL-13, IL-12, IL-10, IL-2R, IL-17, HGF-35, TNF-alpha, IFN-alpha, IFN-gamma, CCP1/MCp1, MIP-1B, MIPI-1A, VEGF, FGF-B, FGF-12, MIG-63, GMCSF27, IP-10, G-CSF, and RANTES, Life Technologies) according to manufacturer’s instructions, which included room temperature incubation, with agitation, of samples with antibody beads in 96-well plates; room temperature incubation with biotinylated detector antibodies; and room temperature incubation with streptavidin-R-pycoerythrin. Between incubation steps were decanting and washing steps, using a magnetic plate separator. Finished plates were scanned using a Luminex Multiplex Bead Array system (Bio-Plex 200, Bio-Rad, Hercules, CA). Standards provided with the Cytokine panel were serially diluted to 7 concentrations according to manufacturer’s protocols, and included in the 96-well plate with tumor samples for processing. Specimens were run in duplicate and the ranges of detected values and coefficients of variation (CV) for IL-17, GMCSF27, IL-10 and IL-15 were 1.47-198.1 pg/ml, %CV 0–110.96%; 1.35-215.12 pg/ml %CV 0.35-57.47; 2.53-327.55 pg/ml, %CV 0–27.08%; and 50.94-796.76pmg/ml, %CV 0.23-26.44%, respectively. Additional file 1: Table S1 shows ranges of detection values for all 30 cytokines/chemokines analyzed, and the number of samples for which cytokines/chemokines were under the detection limits. For IL-10 and GMCSF all samples were within detection limits. For IL-17, there were 2 samples under the detection limits, which were excluded from analysis. And for IL-15, 10 samples were under detection limits and also excluded from analysis (Additional file 1: Table S1).

### Statistical analysis

The goal of the analysis was to determine if cytokines and chemokines examined varied significantly based on infection with HPV 16 or 18 or other genotypes. All women infected with HPV 16 or 18, regardless of infection with other HPV genotypes were designated to have HPV 16/18. All women with no detectable HPV16 or 18 were designated ‘other’. One woman with no detectable HPV was excluded from analysis. T-tests were used to examine if there were differences in cytokine/chemokine abundance between women infected with HPV16/18 and those infected other HPV genotypes. The natural logarithm of cytokines/chemokines values was used in all the analyses. Of the 30 cytokines and chemokines examined, those significantly different (p < 0.05) were further evaluated in generalized linear regression models (GLM) to adjust for potential influence of hormonal contraceptives use, parity and age; among the 31 cancer cases analyzed, 2 reported smoking and all were multiparous. Regarding lifetime sexual partners, as previously described [8], cancer cases reported either 1 or 2 sexual partners. Only 3 women reported 3 lifetime sexual partners and none reported more than 3 lifetime sexual partners. Excluding the three cases with 3 lifetime sexual partners and the 2 smokers from the statistical models did not alter the findings. Four women were also infected with HIV-1; after excluding them from analysis, results remained unchanged. All p-values reported are two-sided. Statistical analyses were conducted using SAS 9.2 (SAS Institute, Cary, NC).

## Results

Table 1 summarizes the distributions of established risk factors for ICC and infection with human papillomavirus, dichotomized by HPV genotype 16 or 18 vs. others. We found that among the 31 cases of ICC, women with genotypes HPV 16/18 were younger (mean age = 51.86 years, sd = 11.34) compared to women who did not harbor HPV 16/18 (mean age = 60.11 years, sd = 12.17) (p = 0.041). The mean number of HPV genotypes among the group with HPV16 and/or 18 was 3.05 (sd = 0.32), the mean number of HPV genotypes among women with no HPV 16 and/or 18 was 1.44 (sd = 1.74) (p = 0.992). There were no differences in oral contraceptive use between groups (p = 0.397). All women were multiparous and only 2 (among those infected with HPV 16 and/or 18) of the 31 reported smoking cigarettes.

**Table 1:** **Characteristics of 31 study participants with ICC by HPV infection**

	**HPV 16/18 (−) n = 9**	**HPV 16/18 (+) n = 22**	**p-value**
Age*	60.11 (12.17)	51.86 (11.34)	0.041
	(44–82)	(29–75)	
**Smoking**			0.350
***Yes***	0	2 (9.1)	
***No***	9 (100)	20 (90.91)	
Number of HPV types*			0.992
	1.44 (1.74)	3.05 (.32)	
	(0–5)	(1–6)	
**Oral Contraceptive Use**			0.397
***Yes***	3 (33.3)	11 (50)	
***No***	6 (66.7)	11 (50)	

**ICC cases with and without HPV 16/18 infection.** Of 31 ICC cases, 22 had HPV 16/18, which were statistically younger than ICC cases with no HPV16/18 infection (p=0.041). No differences were observed in number of HPV types and oral contraceptive use between ICC cases. Only 2 women reported smoking.

*For age and number of HPV types, mean, SD and range are reported.

For all other variables, frequency and percentages are reported.

The prevalence of HPV16 or 18 was comparable in the overall sample of 40 representing all ICC cases previously reported [8], and in this sub-sample of 31 in which cytokines were successfully measured (73%, 95% CI = 65% to 81%). We found that in this population of African women, and as previously described [8], the 31 cancer samples had multiple infections with high-risk types HPV 16 (73%), HPV 18 (21%), HPV 35 (33%), HPV 45 (30%), HPV 31 (18%), HPV 52 (15%), HPV 33 (9%), HPV 58 (6%), and HPV 68 (3%) [8]. HPV16 and 18 were present in 22 ICC cases out of 31.

**Figure 1:**
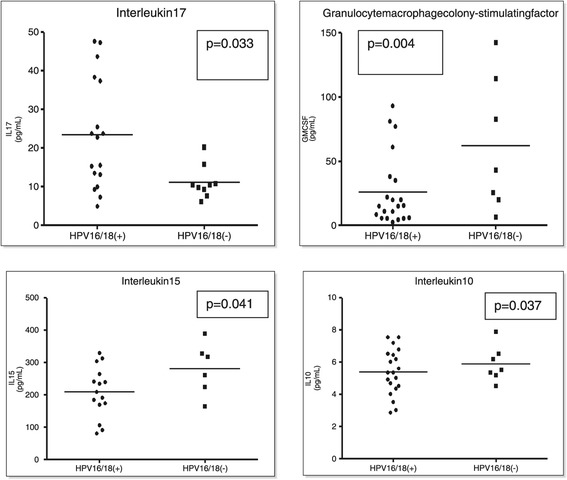
**Cytokine concentrations in ICC samples infected with HPV 16/18 vs. ICC samples without HPV 16/18.** P-values from generalized linear models, after adjusting for age, parity and hormonal contraceptive use.

Among a panel of 30 cytokines and chemokines including IL-15, IL-4, IL-2, IL-1RA, IL-1B, IL-5, IL-6, IL-7, IL-8, EGF, IL-13, IL-12, IL-10, IL-2R, IL-17, HGF-35, TNF-alpha, IFN-alpha, IFN-gamma, CCP1/MCp1, MIP-1B, MIPI-1A, VEGF, FGF-B, FGF-12, MIG-63, GMCSF27, IP-10, G-CSF, and RANTES, measured in homogenized cervical cancer tissues, we detected a trend for differences between cytokines/chemokines and HPV16/18 status; in general, pro-inflammatory cytokines/chemokines were higher in women infected with HPV 16/18 compared to those infected with HPV genotypes other than HPV16/18. However only four cytokines varied significantly between ICC harboring HPV 16 or 18 vs. ICC harboring non HPV 16 or 18 (Figure 1).

We found that women infected with HPVs other than 16 or 18 had lower concentrations of IL-17 when compared to those who carried HPV 16 and/or 18 (p = 0.0006) after adjusting for oral contraceptives and parity; these findings were significant even after applying the stringent Bonferroni correction (0.05/30 = 0.0017). However, further adjusting for age somewhat weakened these associations although they remained statistically significant (p = 0.033) (Figure 1). Cancer tissue from women not infected with HPV 16 or 18 had higher concentrations of GMCSF (p = 0.004), IL10 (p = 0.037), and IL15 (p = 0.041), when compared to those of women infected with HPV16/18 (Figure 1), after adjusting for age, parity and hormonal contraception. IL-6 was also higher in cancer samples with HPV other than 16 and 18, however the differences were not statistically significant (data not shown). Repeating these analyses excluding the two smokers and the 4 cases infected with HIV-1 did not alter these findings. No other chemokine/cytokine significantly differed between cancer cases that harbored HPV16/18 and those that harbored other HPV genotypes.

## Discussion

Although 70% of invasive cervical cancer cases are thought to be caused by HPV 16 and 18, recent data suggest that even in severe cervical intraepithelial neoplasia, women of African descent are two times less likely to be infected with HPV 16 and 18 [5-7]. This observation suggests that invasive cervical cancer may be driven by a more diverse set of HPV genotypes in this population, which has important implications in screening for precancerous lesions [7]. Reasons for these differences are still unclear, although previous findings have been complicated by the use of cervical scrapes, which include cancer cells and normal cells distal to the tumor. Previous studies have also been difficult to assess because of a higher genetic admixture found in African Americans [21]. But most importantly, there are few studies that included cervical tumor tissue.

In these analyses, we tested the hypothesis that immune response may be different in individuals infected with HPV other than 16 and 18, by comparing the levels of cytokines and chemokines in cervical tissues of women infected with HPV 16/18 to those infected with other HPV genotypes in an African population. Our key finding was that there were significantly lower levels of IL-17 and higher levels of IL-10, IL-15 and GMCSF in women who carried HPV genotypes other than HPV16 and 18. These data are consistent with the hypothesis that different HPV genotypes may elicit a different immune response.

These data extend previous work by including a larger number of cytokines. These data also support the hypothesis that infection with HPV genotypes other than 16 and 18 is associated with an immune response different from that triggered by HPV 16 and 18, and suggest that women infected with non-HPV16/18 genotypes (predominantly of African descent) may be more likely to progress from HPV infection to ICC.

Our finding that pro-inflammatory IL-17 levels in cervical tissues were significantly higher in women infected with HPV 16 and/or 18 is in agreement with other studies that reported an association between increased IL-17 levels and cervical cancer cell growth both in human and in animal studies [22,23]. Furthermore, the evidence that IL-17 expression was lower in ICC tissue that did not harbor HPV16 or 18 suggests that other cytokines, which may include GMCSF, IL-10 and IL-15, may trigger an immunosuppressive response that is permissive for development of ICC when the cervical tissue is infected with other non-HPV16 or 18 HR-HPV genotypes. The sample size was too limited for us to model this possibility.

Previous studies have reported on the dual biological function of IL-10 as an anti-inflammatory (potentially cancer-promoting) and anti-angiogenic (potentially cancer-inhibiting) agent, which reflects the conflicting data in cervical cancer [24]. Increased expression of IL-10 was shown to be associated with decreased risk of CIN2/3 lesions, when controlled for potential confounders [25]. However, Syrjänen et al., 2009 [26] found that IL-10 up-regulation in baseline biopsies was most consistently associated with high-grade CIN, but the expression profile of this cytokine was unrelated to HR-HPV genotypes or viral load, and was not a predictor of viral outcome and disease progression in a longitudinal setting. After accounting for high-risk-HPV, age, and use of oral contraceptive, they found IL-10 was an independent covariate of CIN2/3, suggesting that this immunosuppressive cytokine might play an important role in creating a milieu that favors progressive cervical disease [26]. None of these studies however analyzed the data by HPV 16 or 18 vs. other HR-HPV genotypes, which could add another dimension to the immune response elicited by IL-10 towards progression from CIN2/3 to ICC.

Our study has several limitations including the small number of ICC cases examined and thus we cannot draw definitive conclusions. Even though we had data on HIV-1 infection, data on Th2/Th1 response, haematological evidence of autoimmune disorders and other immunological records were not available, thus while unlikely, we cannot rule out that some of the differences found could be due in part to immune disorders, as well as the fact that women infected with HPV 16/18 were on average infected with a higher number of HPV genotypes than the group without HPV 16/18. A key question that remains to be answered, if interventions aimed at reducing disparities are to be effective, is whether or not race/ethnicity differences observed are the consequence of a higher propensity for infection with specific HPV type variants in different race/ethnic groups. If so, this may suggest differences in immunological response to HPV or co-infections. However, our cohort included only African women whose cytokine profile may not be comparable to that in ICC samples of white women. Although, we had 31 ICC cases with confirmed HPV infection obtained directly from the lesion.

## Conclusions

These findings indicate that distinct HR-HPV genotypes may induce different immune responses that facilitate progression from CIN to ICC, which may vary by race/ethnicity. Such inflammatory markers could be useful in risk stratification to discriminate women likely to progress among those with abnormal cytological findings and HPV infection. Future studies in multiethnic cohorts are needed to confirm this hypothesis.

## Additional file

Additional file 1: Table S1. Detection ranges of the 30 cytokines/chemokines studied. In column three the number of samples with cytokines/chemokines under the detection limit, are shown. Those samples were excluded from analysis (*). Seven RANTES samples with detection ranges over the limit were also excluded from analysis.
