# Supplementary material for: IL-10, IL-15, IL-17, and GMCSF levels in cervical cancer tissue of Tanzanian women infected with HPV16/18 vs. non-HPV16/18 genotypes
Source: Infect Agent Cancer. 2015 Mar 20;10:10. doi: 10.1186/s13027-015-0005-1 (PMC4373111; doi:10.1186/s13027-015-0005-1)
Supplement: Additional file 1: Table S1. — Detection ranges of the 30 cytokines/chemokines studied. In column three the number of samples with cytokines/chemokines under the detection limit, are shown. Those samples were excluded from analysis (*). Seven RANTES samples with detection ranges over the limit were also excluded from analysis. [file 13027_2015_5_MOESM1_ESM.docx]

**Additional Table S1.** **Detection ranges of the 30 cytokines/chemokines studied.** In column three the number of samples with cytokines/chemokines under the detection limit, are shown. Those samples were excluded from analysis (*). Seven RANTES samples with detection ranges over the limit were also excluded from analysis.

| **Cytokine/chemokines** | **Detection Range (pg/ml)** | **N^o^ Samples under/over detection limits** |
| --- | --- | --- |
| IFN-γ | 15.7-11346 | 17* |
| IL-4 | 58.2-43356 | 0 |
| IL-12 | 15.5-11223 | 0 |
| TNF-α | 9.3-6716 | 0 |
| IL-5 | 6.9-5089 | 0 |
| IL-13 | 26.4-18370 | 1* |
| MCP-1 | 20.3-14898 | 0 |
| VEGF | 7.9-6268 | 0 |
| FGF-β | 5.1-1147.2 | 0 |
| IL1-β | 12.2-8864 | 1* |
| IL-6 | 6.8-5020 | 0 |
| **IL-17** | **30.3-21618** | **2*** |
| MIG | 21.6-4326 | 0 |
| **GMCSF** | **23.5-14588** | **0** |
| IL-2 | 15.1-10899 | 1* |
| **IL-10** | **28.7-19069** | **0** |
| IP-10 | 5.6-3648 | 10* |
| MIP-1α | 25.2-16524 | 0 |
| GCSF | 82.4-58309 | 0 |
| RANTES | 17.6-10526 | 7* **(over the limit)** |
| Eotaxin | 4.9-4032 | 0 |
| **IL-15** | **31.3-29269** | **10*** |
| EGF | 10.4-7236 | 18* |
| HGF | 13.7-11268 | 1* |
| IFN-α | 18.6-12324 | 1* |
| IL-1Rα | 96.7-57548 | 0 |
| IL-7 | 9.0-6663 | 3* |
| IL-2R | 29.9-22924 | 1* |
| IL-1 | 14.8-10517 | 8* |
| MIP-1β | 11.1-8277 | 1* |
| *Excluded from analysis |  |  |
